# Effects of *Fructus Aurantii* Extract on Growth Performance, Nutrient Apparent Digestibility, Serum Parameters, and Fecal Microbiota in Finishing Pigs

**DOI:** 10.3390/ani14243646

**Published:** 2024-12-17

**Authors:** Haiqing Gan, Qian Lin, Yecheng Xiao, Qiyu Tian, Chao Deng, Renjie Xie, Hongkun Li, Jiajie Ouyang, Xingguo Huang, Yang Shan, Fengming Chen

**Affiliations:** 1 Hunan Agricultural Product Processing Institute, Hunan Academy of Agricultural Sciences, Changsha 410125, China; ghq0619123@163.com (H.G.);; 2 College of Animal Science and Technology, Hunan Agricultural University, Changsha 410128, China; 3 Institute of Bast Fiber Crops, Chinese Academy of Agricultural Sciences, Changsha 410205, China; 4 Hunan Biological Electromechanical Vocational Technical College, Changsha 410127, China; 5 Hunan Provincial Key Laboratory of the Traditional Chinese Medicine Agricultural Biogenomics, Changsha Medical University, Changsha 410219, China

**Keywords:** finishing pigs, *Fructus Aurantii* extract, nutrient apparent digestibility, serum parameters, fecal microbiota

## Abstract

This study investigated the effects of *Fructus Aurantii* extract (FAE) on growth performance, nutrient apparent digestibility, serum parameters, fecal microbial composition, and short-chain fatty acids (SCFAs) in finishing pigs. In total, 75 Duroc × Landrace × Yorkshire pigs (equally divided by sex), with an initial body weight of 79.49 ± 4.27 kg, were randomly assigned to three treatment groups. The pigs were fed either a basic diet (CON) or a basal diet supplemented with 500 mg/kg of FAE (FAE500) and 1000 mg/kg of FAE (FAE1000). The FAE1000 group exhibited a significantly higher final body weight (FBW) (*p* < 0.05), and the average daily feed intake (ADFI) showed an increasing tendency in the FAE500 and FAE1000 groups (*p* = 0.056) compared to the CON group. Additionally, the inclusion of FAE resulted in the significantly higher apparent digestibility of crude ash (Ash), gross energy (GE), and crude protein (CP) (*p* < 0.05), with a tendency to the increased digestibility of dry matter (DM) (*p* = 0.053). Dietary FAE supplementation led to elevated serum levels of reduced glutathione (GSH) and decreased levels of serum L-lactic dehydrogenase (LDH), along with a tendency to increase serum glucose (GLU) levels (*p* = 0.084). The FAE500 group demonstrated higher serum concentrations of motilin (MTL) and gastrin (GAS) (*p* < 0.05), and a tendency for reduced serum glucagon-like peptide-1 (GLP-1) level (*p* = 0.055) compared to the CON group. Furthermore, alpha diversity analysis revealed that the FAE500 group significantly increased the Chao 1 and Observed_species indexes (*p* < 0.05). Similarly, beta diversity analysis indicated that FAE feeding altered the fecal microbial structure (*p* = 0.083). Notably, compared with the control group, *CF231*, *Pediococcus*, and *Mogibacterium* displayed higher relative abundance in the feces of the FAE500 group, whereas Tenericutes showed a reduction in relative abundance (*p* < 0.05). Additionally, the relative abundance of Tenericute was negatively correlated with the digestibility of DM, GE, Ash, and CP (*p* < 0.05). Serum MTL and GAS levels correlated positively with the *Coprococcus*, *Dorea*, *Pediococcus*, and *Mogibacterium* relative abundances (*p* < 0.05). Collectively, dietary FAE supplementation could enhance growth performance by boosting beneficial bacteria in feces, stimulating gastrointestinal hormone secretion, and improving nutrient digestibility.

## Simple Summary

*Fructus Aurantii* (FA) is rich in flavonoids, which can effectively promote digestion and improve intestinal health, possessing a strong potential to serve as an alternative to antibiotics. In this experiment, the addition of *Fructus Aurantii* extract increased the relative abundance of beneficial bacteria in feces, stimulated the secretion of gastrointestinal hormones, improved digestion ability and growth performance.

## 1. Introduction

Antibiotic growth promoters (AGPs) have been prohibited in animal feeds in many countries to mitigate the impact of resistance and other adverse production on animals and the environment [1]. Natural plants exhibit a range of biological activities, low toxicity and cost-effectiveness, making them a potential substitute for antibiotics [2]. *Fructus Aurantii* (FA), derived from the dried unripe fruit of *Citrus aurantium* L. and its cultivars [3], contains key components such as flavonoids, volatile oils, alkaloids, and coumarins in its extract (FAE). Numerous studies have demonstrated that FA possesses extensive pharmacological properties. It has been shown to treat dyspepsia and enhance gastrointestinal function, alleviate chest pain, and treat organ prolapse [4]. Additionally, FA can mitigate oxidative stress by scavenging free radicals [5] and up-regulating the expression of antioxidant enzymes by regulating nuclear factor erythroid 2-related factor 2/antioxidant response element (Nrf2/ARE) signaling pathways [6]. Furthermore, FAE exhibits anti-inflammatory properties by modulating various signaling pathways, thereby influencing the expression of inflammatory mediators, including nuclear factor kappa-B (NF-κB) and mitogen-activated protein kinase (MAPK) [7,8]. The flavonoids present in FA may interact with gut flora to positively affect the intestinal health of the host [9]. Notably, flavonoids are the most representative components of FA. Several studies have indicated that flavonoids enhance growth performance and meat quality in livestock and poultry, attributed to their antioxidant, antibacterial, and digestive properties [10,11]. Consequently, these findings suggest that FAE may improve intestinal health and growth performance in pigs. However, previous research on FA has predominantly focused on human health, with less emphasis on livestock. This study aims to investigate the effects of FAE on growth performance, nutrient apparent digestibility, serum parameters, and fecal microbiota composition in finishing pigs.

## 2. Materials and Methods

Procedures for the care and handling of animals required for this experiment were approved by the Hunan Agricultural University Institutional Animal Care and Use Committee (Permission No. 2020A34).

### 2.1. Fructus Aurantii Extract

FAE was supplied by Lianyuan Kanglu Biotechnology Co., Ltd., (Lou Di, China) in powder form (flavonoid > 40%).

### 2.2. Experimental Animals and Rearing

A total of 75 Duroc × Landrace × Yorkshire pigs, evenly divided by sex, with an initial body weight of 79.49 ± 4.27 kg, were randomly assigned to three treatments with five replicates of five pigs each. The treatments included a basal diet (CON), and basal diets supplemented with FAE at concentrations of 500 mg/kg (FAE500) and 1000 mg/kg (FAE1000). All pigs were housed in a clean environment throughout the experiment. The duration of the experiment was 35 days. The composition of the basic experimental diet (Table 1) adhered to the recommendations set forth by the NRC (NRC-2012). The trial was conducted at the Kelikang Liuyang Puji Farm located in Changsha, Hunan. The pigs were fed ad libitum and ensured unrestricted access to water.

### 2.3. Performance Measurement and Sampling

All of the pigs’ body weight measurements were carried out at the beginning and end of the experiment following an overnight fasting period of 12 h. Additionally, feed intake was recorded per pen daily to calculate the average daily gain (ADG), average daily feed intake (ADFI), and feed conversion ratio (FCR). Feed samples were collected weekly, mixed, and stored at −20 °C until chemical analysis was performed. During days 32 to 35 of the experiment, fecal samples were collected from each replicate, placed in sample bags and 15 mL Eppendorf tubes, and stored at −20 °C and −80 °C. Blood samples (10 mL per pig) were collected from the jugular vein of 15 pigs (one pig from each replicate) at 8:00 a.m. on day 35 after the overnight fasting period. These samples were immediately frozen at −80 °C post-centrifugation (3500× *g* at 10 °C for 10 min) for subsequent laboratory analysis.

### 2.4. Chemical Analysis

Nutrient apparent digestibility was determined by the method described by Schiavone et al. [12]. The feed and fecal samples were desiccated at 65 °C in an oven, rehydrated for 72 h at room temperature, and subsequently crushed to pass through a 1 mm sieve. The moisture content (GB/T 6435-2014) (https://www.chinesestandard.net/PDF.aspx/GBT6435-2014 accessed on 4 November 2024), crude ash (GB/T 6438-2007) (https://www.chinesestandard.net/PDF.aspx/GBT6438-2007 accessed on 4 November 2024), crude protein (CP) (GB/T 6432-2018) (https://www.chinesestandard.net/PDF.aspx/GBT6432-2018 accessed on 4 November 2024), crude fiber (CF) (GB/T 6434-2022) (https://www.chinesestandard.net/PDF.aspx/GBT6434-2022 accessed on 4 November 2024), and ether extract (EE) (GB/T 6433-2006) (https://www.chinesestandard.net/PDF.aspx/GBT6433-2006 accessed on 4 November 2024) were measured according to the national standards of the People’s Republic of China. The dry matter (DM) content of each sample was calculated after drying to a constant weight in a constant temperature oven at 105 °C. An automatic calorimeter (SDAC6000, Hunan Sundy Technology Co., Ltd. Changsha, China) was employed to determine the gross energy (GE). The apparent digestibility of nutrients was assessed using the endogenous indicator approach, with acid-insoluble ash (AIA) serving as the endogenous indicator.

### 2.5. Serum Parameter Analysis

The measurement of alanine aminotransferase (ALT), alkaline phosphatase (ALP), total protein (TP), globulin (GLB), blood glucose (GLU), total cholesterol (TC), aspartate aminotransferase (AST), lactate dehydrogenase (LDH), albumin (ALB), blood urea nitrogen (BUN), triglyceride (TG), high-density lipoprotein cholesterol (HDL-C), and low-density lipoprotein cholesterol (LDL-C) was performed using the Kehua serum automatic biochemical analyzer (Excellence 450, Shanghai Kehua Experimental System Co., Ltd., Shanghai, China) with kits supplied by Shanghai Kehua Bioengineering Co., Ltd., Shanghai, China. In addition, commercial test kits from the Nanjing Jiancheng Bioengineering Institute, Nanjing, China, were employed to assess serum levels of glutathione peroxidase (GSH-Px), malondialdehyde (MDA), reduced glutathione (GSH), catalase (CAT), superoxide dismutase (SOD), and total antioxidant capacity (T-AOC). The serum concentrations of motilin (MTL), gastrin (GAS), and glucagon-like peptide-1 (GLP-1) were measured using ELISA kits from Shanghai Zhuocai Biotechnology Co., Ltd., (Shanghai, China). All procedures were conducted in accordance with the instructions provided by the manufacturers of the kits.

### 2.6. The Microbial Analysis of the Feces

Four fecal samples (12 samples in total) were selected from each group for analysis. The 16S rRNA sequencing was performed by Shanghai Personal Biotechnology Co., Ltd., Shanghai, China. To extract the total genomic DNA from the 12 fecal samples, the M5636-02 magnetic bead soil DNA small extraction kit was utilized. The V3-V4 region of the bacterial 16S rRNA gene was amplified using the primers 338F (5′—ACTCCTACGGGAGGCAGCA—3′) and 806R (5′—GGACTACHVGGGTWTCTAAT—3′). Following amplification, the PCR products were sequenced on the Illumina Miseq platform. The dada 2 technique implemented in QIIME2 (version 2019.4) software was employed to denoise the sequencing data. Each deduplicated sequence generated after quality control was referred to as an amplicon sequence variant (ASV), with a leveling depth set at 95% of the minimum sample sequence quantity. Subsequently, an ASV abundance table was constructed post-leveling. We assessed the alpha diversity, beta diversity, and microbial composition in feces in both the flattened and unflattened ASV abundance tables.

### 2.7. Correlation Analysis of Intestinal Biomarkers with Apparent Nutrient Digestibility and Serum Parameters

Spearman’s correlation analysis was conducted to examine the relationship between the biomarkers of intestinal flora, apparent nutrient digestibility, and serum parameters, utilizing the platform by Shanghai Personal Biotechnology Co., Ltd.

### 2.8. Fecal SCFA Concentration Analysis

Fecal SCFA was quantified using gas chromatography. In brief, 1.0 g of feces was mixed with 5 mL of distilled water and vortexed overnight. The mixture was then centrifuged for 10 min at 4 °C at 10,000× *g*, and the supernatant was collected. Subsequently, 25% (*wt*/*vol*) phosphoric acid was added to the supernatant in a 9:1 (*v*:*v*) ratio, and the mixture was allowed to stand for 3 to 4 h. Following this, the sample was analyzed using gas chromatography (Agilent 8890, Palo Alto, CA, USA) after being filtered through a 0.45 μm membrane.

### 2.9. Statistical Analysis

The means and standard error of the mean (SEM) of the data are presented. All data were analyzed using Duncan’s test, one-way ANOVA, and SPSS v. 24.0 software (IBM-SPSS Inc., Chicago, IL, USA) to determine group differences. A significance level of *p* < 0.05 was established, with *p* < 0.10 indicating a tendency and *p* < 0.001 denoting highly significant results. Bar plots were generated using GraphPad Prism 9 (San Diego, CA, USA).

## 3. Results

### 3.1. Growth Performance

The FAE1000 group exhibited a higher final body weight (FBW) (*p* < 0.05), and the average daily feed intake (ADFI) showed an increasing tendency in the FAE500 and FAE1000 groups (*p* = 0.056) compared to the CON group (Table 2).

### 3.2. The Apparent Nutrient Digestibility

The apparent digestibility of GE, Ash, and CP was significantly higher in the FAE500 and FAE1000 groups compared to the CON group (*p* < 0.05) (Table 3). The apparent digestibility of DM exhibited an increasing trend in the FAE500 and FAE1000 groups relative to the CON group (*p* = 0.053).

### 3.3. Serum Biochemical Indexes

In comparison to the CON group, it was observed that dietary FAE supplementation reduced the serum LDH levels (*p* < 0.05) and in-serum GLU levels (*p* = 0.084) (Table 4).

### 3.4. Serum Antioxidant Indices

Compared to the CON group, dietary FAE supplementation significantly elevated serum GSH content (*p* < 0.05) (Table 5). However, other antioxidant indices did not show substantial differences among the groups (*p* > 0.05).

### 3.5. Serum Hormone Level

**Figure 1:**
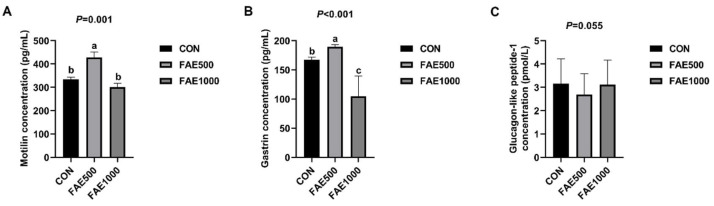
Effects of *Fructus Aurantii* extract (FAE) extract (FAE) on serum hormone level of finishing pigs. (**A**) Motilin concentration. (**B**) Gastrin concentration. (**C**) Glucagon-like peptide−1 concentration. CON = basal diet; FAE500 = basal diet containing 500 mg/kg *Fructus Aurantii* extract; FAE1000 = basal diet containing 1000 mg/kg *Fructus Aurantii* extract; (lowercase letters) values are represented as mean ± SEM, *n* = 4.

Serum MTL and GAS contents were significantly higher (*p* < 0.05) in the FAE500 group compared to the CON group (Figure 1), while serum GLP-1 content in the FAE500 group tended to decrease (*p* = 0.055).

### 3.6. Fecal Bacterial Community

**Figure 2:**
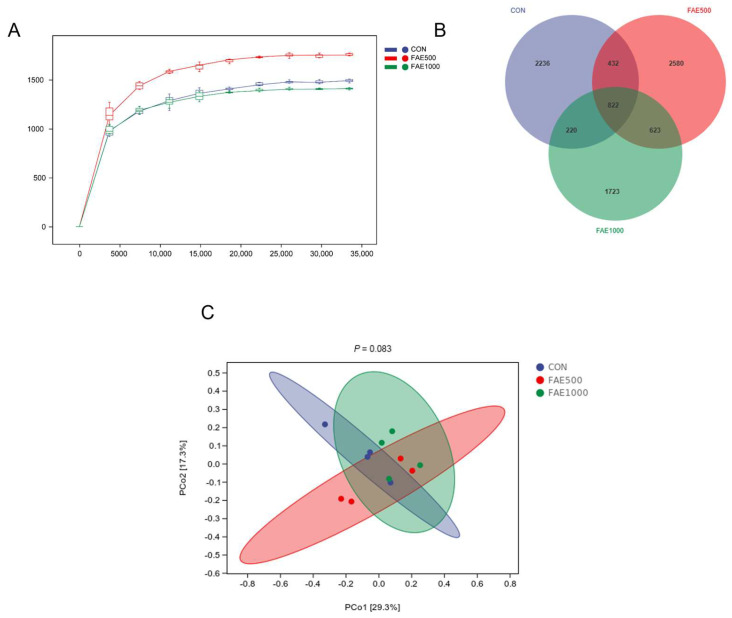
The effect of dietary *Fructus Aurantii* extract (FAE) on microbiota diversity in finishing pigs. (**A**) Rarefaction curve based on the Chao 1 index. (**B**) Venn analysis of amplicon sequence variants (ASV). (**C**) the principal coordinate analysis (PCoA) plot of the bacterial community based on Bray–Curtis distances. Values are represented as mean ± SEM, *n* = 4. ASV, amplicon sequence variant; PCoA, principal coordinates analysis.

We observed alterations in the gut microbiota of finishing pigs, indicated by shifts in both alpha and beta diversity. The rarefaction curve approached a plateau, suggesting that the species present in the current sample were well represented by the sequencing results (Figure 2A). The FAE500 group displayed a greater number of amplicon sequence variants (ASV) compared to the CON and FAE1000 groups (Figure 2B). Furthermore, principal coordinates analysis (PCoA) indicated distinct differences in gut microbiota among the groups (Figure 2C, *p* = 0.083 using PERMANOVA). In the alpha diversity analysis, the FAE500 group showed significant enhancements in the Chao 1 index and observed species index (*p* < 0.05) compared to the CON and FAE1000 groups, although it did not significantly influence Faith_pd, Goods_coverage, Pielou_e, Shannon, and Simpson (Table 6).

**Figure 3:**
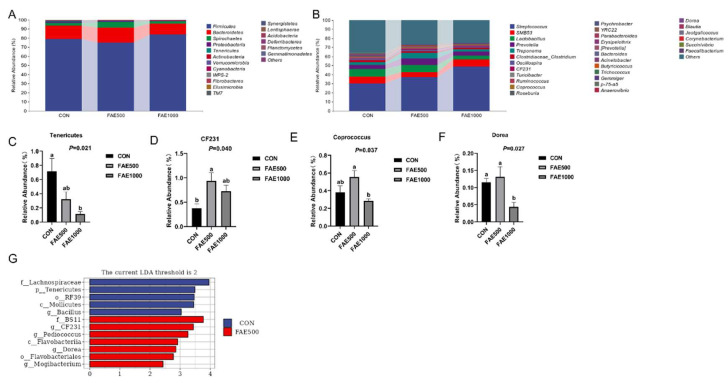
Effects of *Fructus Aurantii* extract (FAE) on the taxon abundance of gut microbiota in finishing pigs (**A**,**B**). It presents the relative abundance of gut microbiota at both the phylum and genus levels, respectively (**C**–**F**). The relative abundance of differential bacteria among groups. The linear discriminant analysis (LDA) value distribution histogram of significantly distinct species (**G**) was derived from Lefse analysis. (lowercase letters) Values are represented as mean ± SEM, n = 4. LDA, linear discriminant analysis; Lefse, linear discriminant analysis—effect size.

The analysis of taxon abundance revealed that dietary FAE influenced the relative abundance of bacteria across various taxon levels, including phylum (Figure 3A) and genus (Figure 3B), when compared to the CON group. The three most prevalent phyla were Firmicutes, Bacteroidetes, and Spirochaetes, which collectively accounted for over 95% of all fecal bacteria. Notably, the FAE1000 group exhibited a reduction in the relative abundance of Tenericutes in comparison to the CON group (*p* < 0.05, Figure 3C). At the genus level, Streptococcus, SMB53, Lactobacillus, Prevotella, Treponema, and Clostridiaceae_Clostridium emerged as the dominant genera, comprising nearly 60% of the total sequences. The FAE500 group demonstrated a significantly higher relative abundance of CF231 compared to the CON group (*p* < 0.05, Figure 3D). Additionally, FAE500 showed increased abundances of Coprococcus and Dorea relative to FAE1000 (*p* < 0.05, Figure 3E,F). LEfSe analysis was employed to identify significantly differential bacterial taxa among the three groups (Figure 3G). The LDA histogram and species taxonomy cladogram indicated that 12 biomarkers were identified in both the CON and FAE500 groups, with 5 in the CON group and 7 in the FAE500 group. However, there was no significant species difference in the FAE1000 group. Tenericutes, Mollicutes, RF39, Lachnospiraceae, and Bacillus were found to be less abundant in the FAE500 and FAE1000 groups compared to the CON group. When comparing the FAE500 group to the CON group, a significant increase in the abundance of Flavobacteria, Flavobacteriales, BS11, CF231, Pediococcus, Mogibacterium, and Dorea was observed.

### 3.7. Fecal SCFA Concentrations

Table 7 indicated that dietary supplementation with FAE had no significant effect on feces SCFA (*p* > 0.05).

### 3.8. Spearman Correlation Analysis

**Figure 4:**
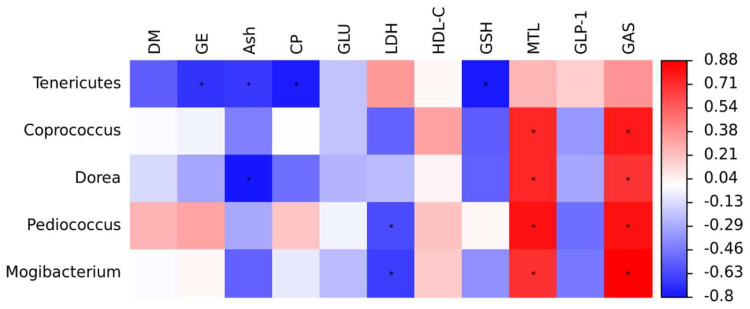
Spearman correlation analysis of gut microbiota with apparent digestibility and serum parameters of finishing pigs. Red and blue colors indicate positive and negative correlations, respectively. Significance is presented as * *p* < 0.05 (*n* = 4).

The relative abundance of Tenericute was negatively correlated with serum GSH concentration, as well as the apparent digestibility of GE, Ash, and CP using the Spearman rank correlation coefficient for significance analysis (Figure 4). Similarly, a negative association was observed between apparent digestibility of ash and Dorea. Furthermore, the relative abundance of *Pediococcus* and *Mogibacterium* were inversely correlated with the serum LDH levels. In contrast, serum levels of MTL and GAS were positively correlated with the relative abundance of *Mogibacterium*, *Dorea*, *Coprococcus*, and *Pediococcus*.

## 4. Discussion

FA exhibits various pharmacological benefits, including improved gastrointestinal motility, protection against gastric ulcers, anti-inflammatory properties, antioxidant effects, anti-tumor capabilities, and the ability to modulate immune responses [4]. Flavonoids are the primary bioactive compounds in FA, including neohesperidin and naringenin as two the major compounds [13]. FA and its constituents play a beneficial role in regulating the secretion of gastrointestinal hormones, which enhances gastrointestinal motility, extends gastric residence time, and improves digestive function [14,15]. Consistently, our study observed that feeding FAE stimulated the secretion of MTL and GAS. Dietary supplementation with 50 mg/kg of naringenin improved body weight gain and feed conversion ratio (FCR) in piglets [16]. Additionally, the inclusion of FAE in the diet notably enhanced ADFI and FBW in finishing pigs, with the FBW for the FAE1000 group being significantly greater than that of the CON group. The growth performance of finishing pigs is likely improved by FAE, which increases gastric motility, promotes the release of gastrointestinal hormones, prolongs the duration that feed remains in the stomach, and enhances digestive capability.

Animal growth performance is also closely associated with the digestive and absorptive capacity of the gastrointestinal tract, and the apparent digestibility of nutrients serves as an indicator of the digestive capacity of pigs [17]. Our findings indicated that feeding FAE improved the apparent digestibility of DM, GE, Ash, and CP compared with the CON group. These results suggest that FAE enhances the utilization of energy and protein in finishing pigs, which aligns with the observed improvements in growth performance. The enhanced digestion and absorption efficiency of feed contribute positively to pigs’ digestive ability and intestinal health, ultimately benefiting their growth [18]. Furthermore, investigation is required to elucidate the specific mechanism by which this improvement may enhance the growth performance of finishing pigs.

Serum biochemical indicators serve as reflections of animal metabolism and health status [19]. LDH is a cytosolic enzyme that enters the bloodstream only when cell membrane integrity is compromised [20]. Notably, we discovered that FAE significantly reduced serum LDH content, suggesting a potential protective effect against cellular damage. HDL-C facilitates the transport of peripheral cholesterol to the liver for metabolic elimination and plays a critical role in regulating cholesterol levels [21]. Our results indicated that HDL-C tended to be lower in the FAE1000 group. Flavonoids derived from *Citrus aurantium* have consistently been reported to lower serum TG, TC, and LDL-C content, boost HDL-C content, and regulate intestinal microbiota in hyperlipidemia rats or mice [9,22]. These findings suggest that the flavonoids in FAE contribute to the regulation of blood lipids [23]. Additionally, studies have shown that butyrate-producing bacteria, such as *Coprococcus*, enhance intestinal health through carbohydrate fermentation products like butyrate, promote the digestion and absorption of nutrients in pigs, and increase serum GLU content [24]. Similarly, fecal microbial analysis in this study revealed a significantly higher relative abundance of butyrate-producing bacteria *Coprococcus* and *Dorea* in the FAE500 group compared to the control group, correlating with the tendency of serum GLU levels. Consequently, the increased relative abundance of butyrate-producing bacteria likely enhances intestinal health, which, in return, may lead to improved nutritional digestion and absorption in finishing pigs, contributing to the observed rise in serum GLU levels.

The stress reaction throughout the production and management processes is one of the most critical elements influencing pig development [25]. These stressors can diminish the antioxidant capacity of pigs, thereby impacting their growth and development [26]. Both enzymatic and non-enzymatic systems play vital roles in mitigating oxidative damage [27]. The antioxidant enzyme system in animals relies on GSH-Px, CAT, and SOD to scavenge oxygen free radicals and prevent oxidative damage. GSH is a vital non-enzymatic antioxidant in cells effectively neutralizing reactive oxygen species [28]. MDA is a significant marker that reflects the levels of lipid peroxidation levels [29]. *Citrus aurantium* L. Cv. Daidai has demonstrated 2,2-diphenyl-1-picrylhydrazyl (DPPH) free radical scavenging activity in HaCaT cells stimulated by H_2_O_2_ and lipopolysaccharides, leading to a notable increase in SOD and GSH content while concurrently reducing reactive oxygen species [30]. The results of the present study suggest that the dietary supplementation of FAE resulted in a significant increase in the serum GSH content in finishing pigs. Feeding FAE also increased serum GSH-Px and SOD activity, but not statistically significant. These findings suggest that FAE can bolster antioxidant enzyme activity and improve antioxidant capabilities by scavenging free radicals.

To elucidate the potential mechanism underlying the enhanced apparent digestibility of nutrients, we measured serum hormone levels and found that feeding 500 mg/kg of FAE significantly increased the levels of MTL and GAS content while tending to decrease GLP-1 content. MTL, a gastrointestinal peptide hormone released by the mucosal cells of the small intestine, may enhance gastrointestinal motility and accelerate gastric emptying [31]. GAS also promotes gastrointestinal motility and stimulates the secretion of gastric acid and pepsin. A study indicates that both high and low dosages of FA substantially elevate serum levels of MTL and GAS in rats with functional dyspepsia, thereby influencing gastrointestinal hormone production, stimulating gastrointestinal motility, and alleviating gastric motility issues [32]. Consequently, feeding 500 mg/kg of FAE can modulate the release of gastrointestinal hormones, enhance gastrointestinal motility, increase gastrointestinal digestion, improve nutrient apparent digestibility, and ultimately enhance growth performance.

FAE contains flavonoids and polyphenols that exhibit low utilization in the gastrointestinal tract, and these compounds typically interact with gut microbiota [33]. In this study, we analyzed the microbial population using the 16S rRNA sequencing technique. The Venn diagram indicated that the FAE500 group had a higher number of unique ASVs compared to the other groups. Both alpha and beta diversity analyses demonstrated that feeding 500 mg/kg of FAE significantly enhanced gut microbiota diversity [34]. The analysis of gut microbiota at the phylum and genus levels across the three groups provides further insights into the mechanisms by which FAE feeding improved growth performance in finishing pigs. The phyla Firmicutes and Bacteroidetes were predominant among the three groups, with Bacteroidetes exhibiting a greater relative abundance in the FAE500 group compared to the others. Tenericutes, a unique class of bacteria that typically lack a cell wall and are either parasitic or commensal to eukaryotic hosts, showed a significant decrease in relative abundance with FAE feeding [35]. Furthermore, Tenericutes exhibited a negative correlation with the apparent digestibility of nutrients in finishing pigs, indicating that they play a critical role in nutrient digestion.

Gut microbiota-derived metabolites serve as crucial mediators between the microbiota and the host, playing a significant role in intestinal health [36]. SCFAs are primarily produced by Firmicutes and Bacteroidetes through the fermentation of indigestible sugars [37]. At the genus level, we observed that beneficial bacteria, including *CF231*, *Coprococcus*, and *Dorea,* were enriched in the FAE group, which is closely associated with SCFA production. *CF231,* belonging to Bacteroidetes, is recognized as a beneficial bacterium. It has been reported that *CF231* abundance is positively correlated with amino acid metabolisms, carbohydrate metabolism, digestive system function, and the production of intestinal metabolites, such as bile acids, SCFAs, and lipid metabolites [38,39]. This suggests that *CF231* plays an active role in cell metabolism and the generation of microbiota-derived metabolites. *Coprococcus* is another beneficial microbe in pigs, contributing positively to health by producing vitamin D and butyrate [40,41]. *Dorea* is known to produce acetate [42], and is also positively correlated with body weight [43]. Interestingly, the present study found no effect of FAE on SCFA levels. Additionally, we discovered that serum MTL and GAS levels were positively correlated with the relative abundance of *Coprococcus* and *Dorea*. This indicates that FAE may enhance the relative population of bacteria capable of generating SCFAs, thereby stimulating the secretion of gastrointestinal hormones and promoting digestive function [44].

LEfSE analysis revealed that *CF231*, *Pediococcus*, *Dorea,* and *Mogibacterium* were significantly enriched in the FAE500 group. *Pediococcus,* a lactic acid bacterium, has been utilized as a feed additive for the production of pediocin [45]. *Pediococcus* enhances intestinal health by inhibiting the growth of pathogenic bacteria and maintaining the balance of intestinal microbiota [46,47]. Previous studies have shown that the relative abundance of Mogibacterium in weaned piglets is positively correlated with SCFA concentrations, which are beneficial for their health [32]. In conclusion, FAE has been found to modify the fecal microbial community composition in finishing pigs which may be linked to increased nutrient apparent digestibility and elevated levels of gastrointestinal hormone release, thereby promoting the growth of finishing pigs.

## 5. Conclusions

In conclusion, dietary FAE supplementation has the potential to enhance growth performance by increasing the abundance of beneficial bacteria in feces, stimulating the secretion of gastrointestinal hormones, and improving nutrient digestibility. These findings indicate that FAE could serve as a potential feed additive to promote growth in finishing pigs.

## Figures and Tables

**Table 1:** Ingredients and nutrient levels of basal diets (%, as-fed basis) ^1^.

Items	Content
Ingredients	
Corn	73.00
Soybean meal	18.00
Wheat bran	5.00
Lysine	0.30
CaHPO_4_	0.50
Limestone	1.09
NaCl	0.40
Phytase 5000 IU	0.04
Premix ^1^	1.67
Total	100.00
Nutrient levels ^2^	
Digestible energy, MJ/kg	14.20
Crude protein	14.31
Total Lysine	1.03
Calcium	0.62
Total phosphorus	0.41

^1^ Premix provides the following per kilogram of diets: VA, 15,000 IU; VD3, 3100 IU; VE, 160 IU; VK3, 3 mg; VB1, 3 mg; VB2, 6 mg; VB6, 5 mg; VB12, 0.03 mg; VC, 250 mg; Niacin, 45 mg; D-pantothenic acid, 9 mg; Folic acid, 1 mg; Biotin, 0.30 mg; Fe (from FeSO4·H_2_O), 170 mg; Cu (from CuSO_4_·5H_2_O), 15 mg; Mn (from MnSO_4_·H_2_O), 80.00 mg; Zn (from ZnSO_4_·H_2_O), 150 mg; I (from KI), 0.90 mg; Se (from Na_2_SeO_3_), 0.20 mg; Mg (from MgO), 69 mg; Co (from CoCl_2_), 0.30 mg. ^2^ Digestible energy was calculated, while the others were measured.

**Table 2:** Effects of *Fructus Aurantii* extract (FAE) on growth performance of finishing pigs ^1^.

Items	CON	FAE500	FAE1000	SEM	*p*-Value
IBW, kg	79.16	79.00	80.32	1.22	0.904
FBW, kg	112.16 ^b^	115.96 ^ab^	117.66 ^a^	0.94	0.036
ADFI, kg/d	2.46	2.54	2.56	0.02	0.056
ADG, kg/d	0.97	1.09	1.10	0.04	0.349
FCR	2.57	2.42	2.34	0.09	0.615

Abbreviations: CON = basal diet; FAE500 = basal diet containing 500 mg/kg *Fructus Aurantii* extract; FAE1000 = basal diet containing 1000 mg/kg *Fructus Aurantii* extract; SEM = standard error of the mean; IBW = initial body weight; FBW = final body weight; ADFI = average daily feed intake; ADG = average daily gain; FCR = feed conversion ratio. ^1^ Values are represented as mean and SEM, *n* = 5. ^a,b^ Within a row, different superscripts mean significant difference (*p* < 0.05).

**Table 3:** The effects of *Fructus Aurantii* extract (FAE) on the apparent digestibility of the nutrients of finishing pigs (%) ^1^.

Items	CON	FAE500	FAE1000	SEM	*p*-Value
DM	79.18	84.05	84.61	1.05	0.053
GE	81.69 ^b^	86.00 ^a^	86.66 ^a^	0.84	0.018
Ash	60.10 ^b^	68.71 ^a^	71.80 ^a^	1.99	0.011
CF	44.38	46.59	49.10	1.22	0.295
CP	83.96 ^b^	89.32 ^a^	88.18 ^a^	0.96	0.039
EE	75.20	78.33	79.24	0.96	0.232

Abbreviations: CON = basal diet; FAE500 = basal diet containing 500 mg/kg *Fructus Aurantii* extract; FAE1000 = basal diet containing 1000 mg/kg *Fructus Aurantii* extract; SEM = standard error of the mean. DM = dry matter; GE = gross energy; CF = crude fiber; CP = crude protein; EE = ether extract. ^1^ Values are represented as mean and SEM, *n* = 5. ^a,b^ Within a row, different superscripts mean significant difference (*p* < 0.05).

**Table 4:** Effects of *Fructus Aurantii* extract (FAE) on serum biochemical indexes of finishing pigs ^1^.

Items	CON	FAE500	FAE1000	SEM	*p*-Value
TP, g/L	67.57	68.14	73.19	1.60	0.285
GLB, g/L	51.33	49.75	43.00	2.34	0.311
ALB, g/L	15.91	18.49	30.34	3.14	0.112
GLU, mmol/L	4.27	4.41	5.01	0.15	0.084
ALT, U/L	74.08	72.39	66.77	3.91	0.756
AST, U/L	34.89	27.11	35.37	2.00	0.255
ALP, U/L	188.93	170.31	157.08	8.61	0.376
LDH, U/L	700.00 ^a^	422.40 ^b^	513.80 ^b^	34.52	0.001
TG, mmol/L	0.38	0.36	0.44	0.02	0.302
TC, mmol/L	2.56	2.61	2.77	0.06	0.397
HDL-C, mmol/L	1.17	1.23	1.08	0.03	0.084
LDL-C, mmol/L	1.30	1.43	1.15	0.06	0.160
BUN, mmoL/L	3.61	5.22	4.88	0.32	0.111

Abbreviations: CON = basal diet; FAE500 = basal diet containing 500 mg/kg *Fructus Aurantii* extract; FAE1000 = basal diet containing 1000 mg/kg *Fructus Aurantii* extract; SEM = standard error of the mean; TP = total protein; GLB = globulin; ALB = albumin; GLU = glucose; ALT = alanine aminotransferase; AST = aspartate aminotransferase; ALP = alkaline phosphatase; LDH = lactate dehydrogenase; TG = triglyceride; TC = total cholesterol; HDL-C = high-density lipoprotein cholesterol; LDL-C = low-density lipoprotein cholesterol; BUN = blood urea nitrogen. ^1^ Values are represented as mean and SEM, *n* = 5. ^a,b^ Within a row, different superscripts mean significant difference (*p* < 0.05).

**Table 5:** Effects of *Fructus Aurantii* extract (FAE) on serum antioxidant indices of finishing pigs ^1^.

Items	CON	FAE500	FAE1000	SEM	*p*-Value
GSH-Px, μmol/L	1386.01	1587.71	1698.82	85.63	0.343
MDA, nmol/mL	4.26	3.99	3.81	0.36	0.890
GSH, mg/L	6.27 ^b^	12.20 ^a^	13.64 ^a^	1.30	0.022
CAT, U/mL	6.74	5.91	6.70	0.29	0.466
SOD, U/mL	11.05	11.64	13.40	0.72	0.394
T-AOC, U/mL	0.85	0.89	0.83	0.04	0.841

Abbreviations: CON = basal diet; FAE500 = basal diet containing 500 mg/kg *Fructus Aurantii* extract; FAE1000 = basal diet containing 1000 mg/kg *Fructus Aurantii* extract; SEM = standard error of the mean; GSH-Px = glutathione peroxidase; MDA = malondialdehyde; GSH = glutathione; CAT = catalase; SOD = superoxide dismutase; T-AOC = total antioxidant capacity; ^1^ values are represented as mean and SEM, *n* = 5. ^a,b^ Within a row, different superscripts mean significant difference (*p* < 0.05).

**Table 6:** Effects of *Fructus Aurantii* extract (FAE) on alpha diversity index of finishing pigs (ug/mL) ^1^.

Items	CON	FAE500	FAE1000	SEM	*p*-Value
Chao1	1490.16 ^b^	1978.50 ^a^	1408.99 ^b^	97.53	0.027
Faith_pd	79.12	87.95	70.41	3.80	0.174
Goods_coverage	0.99	0.99	0.99	0.00073	0.220
Observed_species	1297.88 ^b^	1759.33 ^a^	1287.55 ^b^	85.33	0.027
Pielou_e	0.62	0.63	0.58	0.02	0.526
Shannon	6.42	6.70	5.95	0.25	0.515
Simpson	0.92	0.89	0.85	0.02	0.364

Abbreviations: CON = basal diet; FAE500 = basal diet containing 500 mg/kg *Fructus Aurantii* extract; FAE1000 = basal diet containing 1000 mg/kg *Fructus Aurantii* extract; SEM = standard error of the mean; ^1^ values are represented as mean and SEM, *n* = 4. ^a,b^ Within a row, different superscripts mean significant difference (*p* < 0.05).

**Table 7:** Effects of *Fructus Aurantii* extract (FAE) on short-chain fatty acids content of finishing pigs (ug/mL) ^1^.

Items	CON	FAE500	FAE1000	SEM	*p*-Value
Acetic acid	3212.90	2909.59	2844.74	100.49	0.316
Propionic acid	1237.69	1168.54	1023.69	55.08	0.304
Isobutyric acid	147.14	136.46	118.91	7.17	0.302
Butyric acid	442.10	392.13	343.97	29.89	0.466
Isovaleric acid	228.45	233.31	201.82	9.89	0.431
Valeric acid	162.36	143.34	128.47	10.95	0.511
Total SCFA	5430.64	4983.37	4661.60	161.34	0.143

Abbreviations: CON = basal diet; FAE500 = basal diet containing 500 mg/kg *Fructus Aurantii* extract; FAE1000 = basal diet containing 1000 mg/kg *Fructus Aurantii* extract; SEM = standard error of the mean; SCFA = short-chain fatty acid. ^1^ Values are represented as mean and SEM, *n* = 5.

## Data Availability

The data presented in this study are available upon request from the corresponding author.
